# Complete sequence and comparative analysis of the chloroplast genome of *Plinia trunciflora*

**DOI:** 10.1590/1678-4685-GMB-2017-0096

**Published:** 2017-11-06

**Authors:** Maria Eguiluz, Priscila Mary Yuyama, Frank Guzman, Nureyev Ferreira Rodrigues, Rogerio Margis

**Affiliations:** 1 Programa de Pós-Graduação em Genética e Biologia Molecular, Universidade Federal do Rio Grande do Sul (UFRGS), Porto Alegre, RS, Brazil; 2 Departamento de Biofísica, Centro de Biotecnologia, Laboratório de Genomas e Populações de Plantas, Universidade Federal do Rio Grande do Sul (UFRGS), Porto Alegre, RS, Brazil

**Keywords:** Jaboticaba, Myrtaceae, chloroplast genome, next-generation sequencing

## Abstract

*Plinia trunciflora* is a Brazilian native fruit tree from the Myrtaceae family, also known as jaboticaba. This species has great potential by its fruit production. Due to the high content of essential oils in their leaves and of anthocyanins in the fruits, there is also an increasing interest by the pharmaceutical industry. Nevertheless, there are few studies focusing on its molecular biology and genetic characterization. We herein report the complete chloroplast (cp) genome of *P. trunciflora* using high-throughput sequencing and compare it to other previously sequenced Myrtaceae genomes. The cp genome of *P. trunciflora* is 159,512 bp in size, comprising inverted repeats of 26,414 bp and single-copy regions of 88,097 bp (LSC) and 18,587 bp (SSC). The genome contains 111 single-copy genes (77 protein-coding, 30 tRNA and four rRNA genes). Phylogenetic analysis using 57 cp protein-coding genes demonstrated that *P. trunciflora, Eugenia uniflora* and *Acca sellowiana* form a cluster with closer relationship to *Syzygium cumini* than with *Eucalyptus*. The complete cp sequence reported here can be used in evolutionary and population genetics studies, contributing to resolve the complex taxonomy of this species and fill the gap in genetic characterization.

*Plinia trunciflora* (O.Berg) Kausel, synonym *Myrciaria trunciflora* O.Berg, is a native Brazilian tree that belongs to the Myrtaceae family and is widely distributed in the southern and southeastern areas of Brazil (Sobral *et al.*, 2012). Among all identified *Plinia* sp. species, *P. cauliflora* (DC.) Berg (synonym *M. cauliflora* (Mart.) O.Berg), *P. jaboticaba* (Vell.) Berg (synonym *M. jaboticaba* O.Berg) and *P. trunciflora* are endemic to Brazil. All of these species produce a similar grape-like edible fruit, known as jaboticaba, which presents a sweet jelly-like white pulp covered by a purple peel. Jaboticaba (*P. trunciflora*) has attracted attention because of its significant levels of phenolic compounds associated with health benefits, such as antidepressant and antioxidant effects and the prevention of neurodegenerative diseases and diabetes (Stasi and Hiruma-Lima, 2002; Sacchet *et al.*, 2015). These benefits have largely been attributed to the capacity of these compounds to prevent or reduce oxidative stress. Additionally, jaboticaba (*P. trunciflora*) is largely consumed fresh or used to make jellies, juices, wines, spirits and vinegar (Balerdi *et al.*, 2006).

Despite the nutritional and productive recognized importance of this species, the taxonomic classification is still controversial. This is mostly so because it is based on morphological evaluation of the trees, fruits and seeds, regarding physical, chemical, physicochemical, and germinal characters that have shown the existence of variability (Guedes *et al.*, 2014). Therefore, molecular studies are needed to better clarify the phylogenetic relationships among the species from this genus.

The chloroplast (cp) genome is a circular molecule of double-stranded DNA that consists of four distinct regions, a large and a small single copy region (LSC and SSC, respectively) separated by two inverted repeat regions (IRa and IRb). Despite the high degree of conservation in its structure, gene content and organization, the presence of mutations, duplications and rearrangements of genes make it an attractive option for phylogenetic studies (Costa *et al.*, 2016). In the case of Myrtaceae, there are only few phylogenetic and evolutionary studies based on cp genes (Craven and Biffin 2005; Payn *et al.*, 2007; Biffin *et al.*, 2010; Bayly *et al.*, 2013; Eguiluz *et al.*, 2017; Machado *et al.*, 2017), and there are even less that include the *Plinia* genus (Vasconcelos *et al.*, 2017).

In this study, young leaves from a *Plinia trunciflora* tree harvested in Gravataí, RS, Brazil (latitude (S): 29°51′52″; longitude (W): 50°53′53″) were used to extract total DNA by the CTAB method (Doyle and Doyle, 1990). DNA quality was evaluated by electrophoresis in a 1% agarose gel, and DNA quantity was determined using a NanoDrop spectrophotometer (NanoDrop Technologies, Wilmington, DE, USA). One genomic paired-end library of 100 nt length was generated by Fasteris SA (Plan-les-Ouates, Switzerland) using an Illumina HiSeq2000 platform (Illumina Inc., San Diego, CA, USA). The paired-end sequence reads were filtered against 42 Myrtaceae cp genomes (Table S1) using BWA software with two mismatches allowed (Li and Durbin, 2009). The obtained reads were assembled *de novo* with ABySS software (Simpson *et al.*, 2009). The cp genome scaffolds were orientated using cp genome sequences of *Eucalyptus globulus*, *Eucalyptus grandis* and *Eugenia uniflora* L. using BLASTN (Camacho *et al.*, 2009). A gap region was filled in by Sanger sequencing using primers F: 5’ GGGTTATCCTGCACTTGGAA and R: 3’ TGCTGTCGAAGCTCCATCTA. Genes were annotated using DOGMA (Wyman *et al.*, 2004) and BLAST homology searches. tRNAs (transfer RNA) were predicted using tRNAscan-SE program (Schattner *et al.*, 2005) and confirmed by comparison with the appropriate homologs in *E. globulus*. The circular cp genome map was drawn using OGDRAW online program (Lohse *et al.*, 2007). For the phylogenetic analysis, a set of 57 cp protein-coding sequences (Table S2) from 56 species belonging to Malvids (Eurosids II) (Table S3) were used with *Vitis vinifera* serving as outgroup. Nucleotide sequences were aligned using MUSCLE available in MEGA version 6.0 (Tamura *et al.*, 2013), and a Bayesian tree was generated using MrBayes version 3.1.2 (Ronquist and Huelsenbeck, 2003) with 5,000,000 generations sampled every 100 generations and discarding the first 25% of trees as burn-in, with posterior probability (PP) values for each node. The GTR+I+G nucleotide substitution model determined by MODELTEST version 3.7 (Posada and Crandall, 1998) was used. The phylogenetic tree was rooted and visualized using FigTree software (http://tree.bio.ed.ac.uk/software/figtree/).

A total of 148,824,244 raw Illumina paired-end reads from the *P. truncliflora* nuclear genome were filtered against 42 Myrtaceae cp genomes. The 8,912,157 obtained reads were *de novo* assembled into non-redundant contigs and singletons covering about 99% of the genome (minimum coverage=144 reads, maximum coverage=18,789 reads). Two final large scaffolds were obtained and joined into a cp circular genome using Sanger sequencing. The complete cp genome of *P. trunciflora* is 159,512 bp in size and was submitted to GenBank (accession number: KU318111). The size is similar to that of other Myrtaceae species (Eguiluz *et al.*, 2017; Machado *et al.*, 2017). The cp genome included an LSC region of 88,097 bp, an SSC region of 18,587 bp and a pair of inverted repeats (IRa and IRb) of 26,414 bp each (Figure 1). Coding regions comprise 47.2%, 13.3% correspond to rRNAs and tRNAs, and 39.5% of the genome comprises non-coding regions, including introns, pseudogenes and intergenic spacers (Table 1). In general, all genomic features showed similarity in structure and gene abundance with other Myrtaceae species (Bayly *et al.*, 2013; Eguiluz *et al.*, 2017; Machado *et al.*, 2017). The genome contained 131 genes in total, which includes 111 single-copy genes corresponding to 77 protein-coding genes, 30 transfer RNA (tRNA) genes and four ribosomal genes (rRNA) (Figure 1, Table 1). The *ycf1*, *ycf2* and *ycf15* sequences were annotated as pseudogenes based on the presence of many stop codons in their coding sequences and by comparison with sequences of *E. globulus* and *S. cumini*. Of the 131 genes in *P. trunciflora*, seven of the tRNAs genes and all four rRNA genes occurred within the IR regions and consequently were duplicated (Table 1). The cp genome has 20 intron-containing genes: 12 protein coding genes and six tRNA genes which contain one intron, and the *clpP* and *ycf3* genes that contain two introns each. The *rps12* gene is a trans-spliced gene with the 5’end located in the LSC region and the duplicated 3’end in the IR regions. The *trnK*-*UUU* has 2,529 bp, with the largest intron encompassing also the *matK* gene.

**Figure 1:**
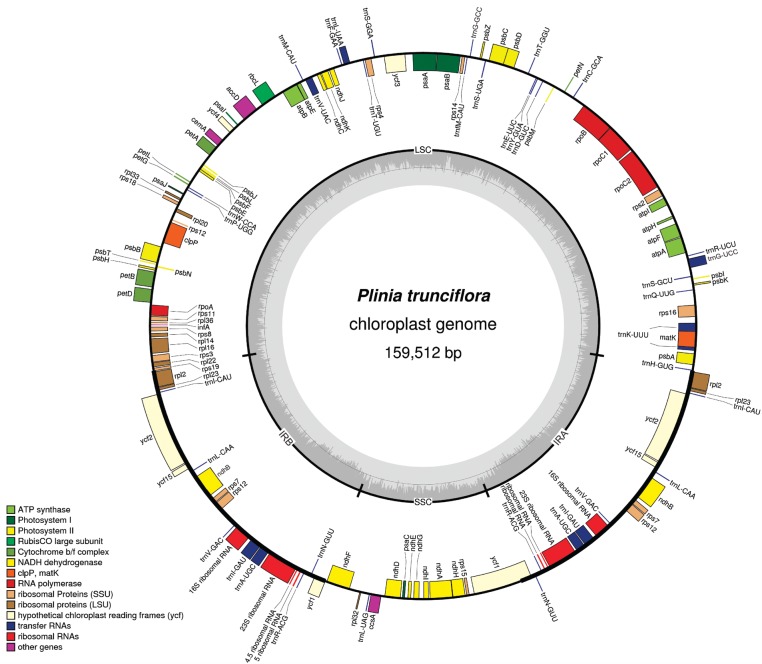
Gene map of the *Plinia trunciflora* chloroplast genome. The structure of the cp genome consists of one large and small single copy (LSC and SSC, respectively) and a pair of inverted repeats (IRa and IRb). Genes drawn inside the circle are transcribed counterclockwise and those outsides are clockwise. Genes belonging to different functional groups are indicated by different tonalities. The darker gray in the inner circle corresponds to GC content, while the lighter gray corresponds to AT content.

**Table 1:** Summary of the *Plinia trunciflora* chloroplast genome characteristics.

Feature	*Plinia trunciflora*
Total cpDNA size	159,512 bp
LSC size (bp)	88,097 bp
SSC size (bp)	18,586 bp
IR size (bp)	26,414 bp
Protein coding regions (%)	60.48%
rRNA and tRNA (%)	13.3%
Introns size (% total)	10.65%
Intergenic sequences and pseudogenes size (%)	28.9%
Number of genes	131 genes
Number of different protein coding genes	77
Number of different tRNA genes	30
Number of different rRNA genes	4
Number of different duplicated genes	16
Pseudogenes	3
GC content (%)	37%

The whole cp genome analysis revealed that the cp genomes of *P. trunciflora* and *E. uniflora* are shorter in comparison to other Myrtaceae, such as *E. globulus*, *E. grandis, E. uniflora* and *S. cumini*, (Figure 2). Despite its size, the total length of introns in *P. trunciflora* (16,972 pb) is the largest in Myrtaceae, e.g. *S. cumini* presents 14,469 bp and the same is observed in *E. globulus* and *E. grandis*. The size of the intergenic spacer located between the IRa/LSC border and the first gene of LSC in *P. trunciflora* is more similar to *Eucalyptus* species than its closer species *E. uniflora* (Figure 2). The comparison of the *ndhK* gene of *P. trunciflora*, with 678 bp, indicated a smaller gene size than that in other plants, such as *E. uniflora* (858 pb), *S. cumini* (855 bp), *E. globulus* (855 bp) and *E. grandis* (853 bp). The same size (678 bp) for this gene is found in *Arabidopsis thaliana*. The effective size of the coding sequence is confirmed by the presence of a thymine in position 53,811 bp in the cp genome from *P. trunciflora* that creates a stop codon and makes this gene shorter than in other Myrtaceae.

**Figure 2:**
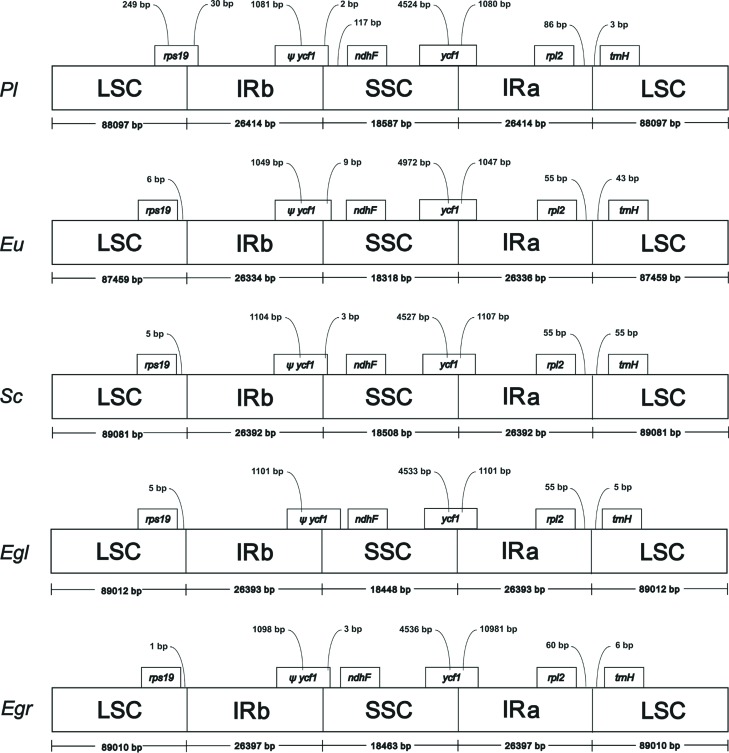
Comparison of the borders of LSC, SSC and IR regions among five chloroplast genomes. Boxes above the main line indicate the predicted genes, while pseudogenes at the borders are shown by Ψ. Variation in *rps19* gene length is displayed at the IRb/LSC borders of *Plinia trunciflora*, *Eugenia uniflora*, *Syzygium cumini*, *Eucalyptus globulus* and *Eucalyptus grandis*, but only in *P. trunciflora*, this gene is located at IRb and LSC regions. This figure is not drawn to scale.

Our phylogeny includes the sister relationship of the orders Brassicales, Malvales and Sapindales and the orders Geraniales and Myrtales. All these results agree with previous studies based on multiple genes or complete cp genomes (Ruhfel *et al.*, 2014). By analyzing the Myrtaceae family clade we showed that *P. trunciflora*, *E. uniflora* and *Acca sellowiana* form a single cluster of Neotropical Myrtaceae, and that this clade has a shorter genetic distance with *S. cumini* than to the Australian Myrtaceae clade (Figure 3). Additionally, our analysis corroborates that *Corymbia gummifera* is paraphyletic in respect to *Angophora*. A previous phylogenetic analysis using certain cp genes (ITS, *matK* and *ndhF*) of Myrtaceae species showed that *Eucalyptus*, *Syzygium*, *Eugenia* and *Myrciaria* (synonym of *Plinia*) form a distinct clade that is consistent with characteristics of the pollen (Thornhill *et al.*, 2012). As can be observed in the Bayesian tree (Figure 3), *Plinia* could be paraphyletic in relation to *Eugenia* and *Acca*, in agreement with the embryo morphology and studies using cp regions that placed *Plinia*, *Myrciaria* and *Siphoneugena* as the emerging “Plinia group” (Lucas *et al.*, 2007). Taxon sampling and phylogenetic methodology could affect the different results. Therefore, additional complete cp genome sequences will help in the comprehension of the relationship among Myrtaceae species.

**Figure 3:**
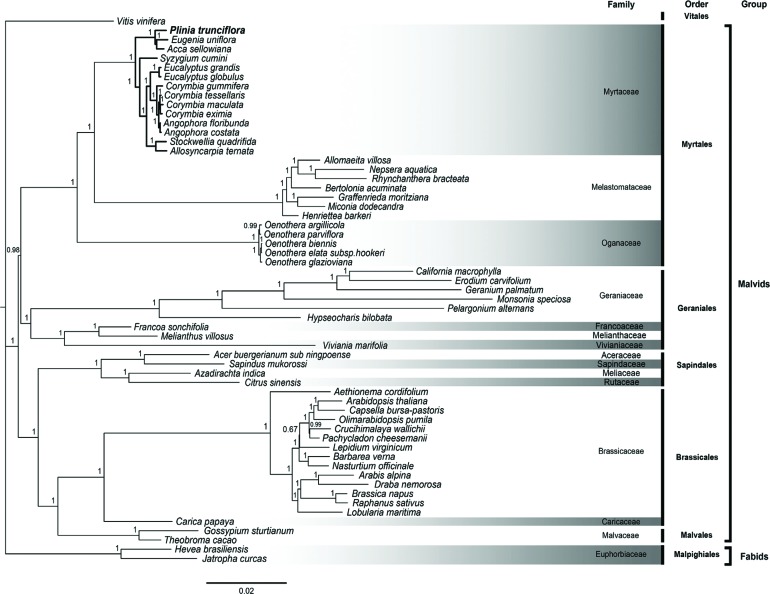
Phylogenetic tree of Eurosids II based on 57 cp protein-coding genes generated by Bayesian method from 56 species. Bold branches indicate the Myrtaceae species. Numbers above each node are posterior probability values. Family, order and clade are also indicated. *Vitis vinifera* was considered as outgroup.

The *Plinia trunciflora* genome represents the first complete cp genome sequence for the genus *Plinia* and shows a set of features that could be further explored for population and phylogenetic studies within this group. Moreover, these data increase the genetic and genomic resources available in Myrtaceae by adding a new strategy of organelle genome assembly.

## Supplementary material

The following online material is available for this article:

Table S1 - List of 42 Myrtaceae chloroplast genomes used in chloroplast genome assembling of *Plinia trunciflora.*

Table S2 - List of 57 chloroplast protein coding genes used in the phylogenetic analysis.

Table S3 - List of 56 plastome sequences of Rosids included in the Bayesian phylogenetic analysis.
