# Supplementary material for: Complete sequence and comparative analysis of the chloroplast genome of Plinia trunciflora
Source: Genet Mol Biol. 2017 Nov 6;40(4):871–6. doi: 10.1590/1678-4685-GMB-2017-0096 (PMC5738614; doi:10.1590/1678-4685-GMB-2017-0096)
Supplement: Supplementary file 1 [file 1415-4757-gmb-1678-4685-GMB-2017-0096-Suppl01.pdf]

Supplementary Material to "Complete sequence and comparative analysis of the chloroplast genome of *Plinia trunciflora*"

Table S1 - List of 42 Myrtaceae chloroplast genomes used in chloroplast genome assembling of *Plinia trunciflora.*

No.	Taxon	GenBank accession number	Study
1	*Allosyncarpia ternata*	KC180806.1	Bayly *et al.* (2013)
2	*Angophora costata*	KC180805.1	Bayly *et al.* (2013)
3	*Angophora floribunda*	KC180804.1	Bayly *et al.* (2013)
4	*Corymbia eximia*	KC180802.1	Bayly *et al.* (2013)
5	*Corymbia gummifera*	KC180800.1	Bayly *et al.* (2013)
6	*Corymbia maculata*	KC180801.1	Bayly *et al.* (2013)
7	*Corymbia tessellaris*	KC180803.1	Bayly *et al.* (2013)
8	*Eucalyptus aromaphloia*	KC180789.1	Bayly *et al.* (2013)
9	*Eucalyptus baxteri*	KC180773.1	Bayly *et al.* (2013)
10	*Eucalyptus camaldulensis*	KC180791.1	Bayly *et al.* (2013)
11	*Eucalyptus cladocalyx*	KC180786.1	Bayly *et al.* (2013)
12	*Eucalyptus cloeziana*	KC180779.1	Bayly *et al.* (2013)
13	*Eucalyptus curtisii*	KC180782.1	Bayly *et al.* (2013)
14	*Eucalyptus deglupta*	KC180792.1	Bayly *et al.* (2013)
15	*Eucalyptus delegatensis*	KC180771.1	Bayly *et al.* (2013)
16	*Eucalyptus diversicolor*	KC180795.1	Bayly *et al.* (2013)
17	*Eucalyptus diversifolia*	KC180774.1	Bayly *et al.* (2013)
18	*Eucalyptus elata*	KC180776.1	Bayly *et al.* (2013)
19	*Eucalyptus erythrocorys*	KC180799.1	Bayly *et al.* (2013)
20	*Eucalyptus globulus*	KC180787.1	Bayly *et al.* (2013)
21	*Eucalyptus globulus* subsp. *globulus*	AY780259.1	Steane (2005)
22	*Eucalyptus grandis*	HM347959.1	Paiva *et al.* (2011)
23	*Eucalyptus guilfoylei*	KC180798.1	Bayly *et al.* (2013)
24	*Eucalyptus marginata*	KC180781.1	Bayly *et al.* (2013)
25	*Eucalyptus melliodora* 1	KC180783.1	Bayly *et al.* (2013)
26	Eucalyptus *melliodora* 2	KC180784.1	Bayly *et al.* (2013)
27	*Eucalyptus microcorys*	KC180797.1	Bayly *et al.* (2013)
28	*Eucalyptus nitens*	KC180788.1	Bayly *et al.* (2013)
29	*Eucalyptus obliqua*	KC180769.1	Bayly *et al.* (2013)
30	*Eucalyptus patens*	KC180780.1	Bayly *et al.* (2013)
31	*Eucalyptus polybractea*	KC180785.1	Bayly *et al.* (2013)
32	*Eucalyptus radiata*	KC180770.1	Bayly *et al.* (2013)
33	*Eucalyptus regnans*	KC180777.1	Bayly *et al.* (2013)
34	*Eucalyptus saligna*	KC180790.1	Bayly *et al.* (2013)
35	*Eucalyptus salmonophloia*	KC180796.1	Bayly *et al.* (2013)
36	*Eucalyptus sieberi*	KC180775.1	Bayly *et al.* (2013)
37	*Eucalyptus spathulata*	KC180793.1	Bayly *et al.* (2013)
38	*Eucalyptus torquata*	KC180794.1	Bayly *et al.* (2013)
39	*Eucalyptus umbra*	KC180778.1	Bayly *et al.* (2013)

No.	Taxon	GenBank accession number	Study
40	*Eucalyptus verrucata*	KC180772.1	Bayly *et al.* (2013)
41	*Stockwellia quadrifida*	KC180807.1	Bayly *et al.* (2013)
42	*Syzygium cumini*	GQ870669.3	Asif *et al.* (2013)

Bayly MJ, Rigault P, Spokevicius A, Ladiges PY, Ades PK, Anderson C, Bossinger G, Merchant A, Udovicic F, Woodrow IE, *et al.* (2013) Chloroplast genome analysis of Australian eucalypts - *Eucalyptus*, *Corymbia*, *Angophora*, *Allosyncarpia* and *Stockwellia* (Myrtaceae). Mol Phylogenet Evol 69:704-716.

Steane DA (2005) Complete nucleotide sequence of the chloroplast genome from the Tasmanian blue gum, *Eucalyptus globulus* (Myrtaceae). DNA Res 12:215-220.

Paiva JA, Prat E, Vautrin S, Santos MD, San-Clemente H, Brommonschenkel S, Fonseca PG, Grattapaglia D, Song X, Ammiraju JS, *et al.* (2011) Advancing Eucalyptus genomics: Identification and sequencing of lignin biosynthesis genes from deep-coverage BAC libraries. BMC Genomics 12:137.

Asif H, Khan A, Iqbal A, Khan IA, Heinze B and Azim MK (2013) The chloroplast genome sequence of *Syzygium cumini* (L.) and its relationship with other angiosperms. Tree Genet Genomes 9:867-877.
