# Supplementary material for: Complete sequence and comparative analysis of the chloroplast genome of Plinia trunciflora
Source: Genet Mol Biol. 2017 Nov 6;40(4):871–6. doi: 10.1590/1678-4685-GMB-2017-0096 (PMC5738614; doi:10.1590/1678-4685-GMB-2017-0096)
Supplement: Supplementary file 2 [file 1415-4757-gmb-1678-4685-GMB-2017-0096-Suppl02.pdf]

Supplementary Material to "Complete sequence and comparative analysis of the chloroplast genome of *Plinia trunciflora*"

Table S2 - List of 57 chloroplast protein coding genes used in the phylogenetic analysis.

Gene	Name	Gene	Name
atpA	ATP synthase CF1 alpha chain	*psbE*	cytochrome b559 alpha subunit
atpB	ATPase beta chain	*psbF*	photosystem II reaction center subunit VI
atpE	ATP synthase epsilon chain	*psbH*	photosystem II reaction center protein H
atpF	ATPase subunit I	*psbI*	photosystem II protein I
atpH	ATPase III subunit	*psbJ*	photosystem II reaction center subunit X
ccsA	cytochrome c biogenesis protein	*psbK*	photosystem II protein K
cemA	heme-binding protein	*psbM*	photosystem II protein M
matK	maturase K	*psbN*	photosystem II reaction center protein N
ndhB	NADH dehydrogenase subunit 2	*psbT*	photosystem II protein T
ndhC	NADH dehydrogenase subunit 3	*rpl14*	ribosomal protein L14
ndhE	NADH dehydrogenase subunit 4L	*rpl16*	ribosomal protein L16
ndhG	NADH dehydrogenase subunit 6	*rpl23*	ribosomal protein L23
ndhI	NADH dehydrogenase 18 kDa subunit	*rpl32*	ribosomal protein L32
ndhJ	NADH dehydrogenase 30 kDa subunit	*rpl33*	ribosomal protein L33
petA	apocytochrome f precursor	*rpl36*	ribosomal protein L36
petB	cytochrome b6	*rpoA*	RNA polymerase alpha chain
petD	cytochrome b6/f complex subunit 4	*rpoB*	RNA polymerase beta chain
petG	cytochrome b6/f complex subunit 5	*rps11*	ribosomal protein S11
petL	cytochrome b6/f complex subunit 6	*rps14*	ribosomal protein S14
petN	cytochrome b6/f complex subunit N	*rps15*	ribosomal protein S15
psaA	photosystem I P700 apoprotein A1	*rps18*	ribosomal protein S18
psaB	photosystem I P700 apoprotein A2	*rps19*	ribosomal protein S19
psaC	photosystem I iron-sulfur center	*rps3*	ribosomal protein S3
psaI	photosystem I subunit VIII	*rps4*	ribosomal protein S4
psaJ	photosystem I subunit IX	*rps7*	ribosomal protein S7
psbA	photosystem II protein D1	*rps8*	ribosomal protein S8
psbB	photosystem II P680 chlorophyll A apoprotein	*ycf3*	photosystem I assembly protein Ycf3
psbC	photosystem II 43 kDa protein	*ycf4*	photosystem I assembly protein Ycf4
psbD	photosystem II protein D2		
