# Supplementary material for: Complete sequence and comparative analysis of the chloroplast genome of Plinia trunciflora
Source: Genet Mol Biol. 2017 Nov 6;40(4):871–6. doi: 10.1590/1678-4685-GMB-2017-0096 (PMC5738614; doi:10.1590/1678-4685-GMB-2017-0096)
Supplement: Supplementary file 3 [file 1415-4757-gmb-1678-4685-GMB-2017-0096-Suppl03.pdf]

Supplementary Material to "Complete sequence and comparative analysis of the chloroplast genome of *Plinia trunciflora*"

Table S3 - List of 56 plastome sequences of Rosids included in the Bayesian phylogenetic analysis.

	Taxon	Family	Order	Accesion number	Study
1	*Aethionema cordifolium*	Brassicaceae	Brassicales	NC_009265.1	unpublished
2	*Arabidopsis thaliana*	Brassicaceae	Brassicales	NC_000932.1	Sato *et al.* (1999)
3	*Barbarea verna*	Brassicaceae	Brassicales	NC_009269.1	unpublished
4	*Brassica napus*	Brassicaceae	Brassicales	NC_016734.1	Hu *et al.* (2011)
5	*Capsella bursa-pastoris*	Brassicaceae	Brassicales	NC_009270.1	unpublished
6	*Carica papaya*	Caricaceae	Brassicales	NC_010323.1	unpublished
7	*Crucihimalaya wallichii*	Brassicaceae	Brassicales	NC_009271.1	unpublished
8	*Draba nemorosa*	Brassicaceae	Brassicales	NC_009272.1	unpublished
9	*Lepidium virginicum*	Brassicaceae	Brassicales	NC_009273.1	unpublished
10	*Lobularia maritima*	Brassicaceae	Brassicales	NC_009274.1	unpublished
11	*Nasturtium officinale*	Brassicaceae	Brassicales	NC_009275.1	unpublished
12	*Olimarabidopsis pumila*	Brassicaceae	Brassicales	NC_009267.1	unpublished
13	*Raphanus sativus*	Brassicaceae	Brassicales	NC_024469.1	Jeong *et al.* (2014)
14	*California macrophylla*	Geraniaceae	Geraniales	JQ031013.1	Weng *et al.* (2014)
15	*Erodium carvifolium*	Geraniaceae	Geraniales	NC_015083.1	Blazier *et al.* (2011)
16	*Francoa sonchifolia*	Melianthaceae	Geraniales	NC_021101.1	Weng *et al.* (2014)
17	*Geranium palmatum*	Geraniaceae	Geraniales	NC_014573.1	Guisinger *et al.* (2011)
18	*Hypseocharis bilobate*	Geraniaceae	Geraniales	NC_023260.1	Weng *et al.* (2014)
19	*Melianthus villosus*	Melianthaceae	Geraniales	NC_023256.1	Weng *et al.* (2014)
20	*Monsonia speciose*	Geraniaceae	Geraniales	NC_014582.1	Guisinger *et al.* (2011)
21	*Pelargonium alternans*	Geraniaceae	Geraniales	NC_023261.1	Weng *et al.* (2014)
22	*Viviania marifolia*	Vivianiaceae	Geraniales	NC_023259.1	Weng *et al.* (2014)
23	*Hevea brasiliensis*	Euphorbiaceae	Malpighiales	NC_015308.1	Tangphatsornruang *et al.* (2011)
24	*Jatropha curcas*	Euphorbiaceae	Malpighiales	NC_012224.1	Asif *et al.* (2010)
25	*Gossypium sturtianum*	Malvaceae	Malvales	NC_023218.1	unpublished
26	*Theobroma cacao*	Malvaceae	Malvales	JQ228389.1	Jansen *et al.* (2011)
27	*Acca sellowiana*	Myrtaceae	Myrtales	KX289887.1	Machado *et al.* (2017)
28	*Allosyncarpia ternate*	Myrtaceae	Myrtales	NC_022413.1	Bayly *et al.* (2013)
29	*Angophora costata*	Myrtaceae	Myrtales	NC_022412.1	Bayly *et al.* (2013)
30	*Angophora floribunda*	Myrtaceae	Myrtales	NC_022411.1	Bayly *et al.* (2013)
31	*Corymbia eximia*	Myrtaceae	Myrtales	NC_022409.1	Bayly *et al.* (2013)
32	*Corymbia gummifera*	Myrtaceae	Myrtales	NC_022407.1	Bayly *et al.* (2013)
33	*Corymbia maculata*	Myrtaceae	Myrtales	NC_022408.1	Bayly *et al.* (2013)
34	*Corymbia tessellaris*	Myrtaceae	Myrtales	NC_022410.1	Bayly *et al.* (2013)
35	*Eucalyptus globulus*	Myrtaceae	Myrtales	KC180787.1	Bayly *et al.* (2013)
36	*Eucalyptus grandis*	Myrtaceae	Myrtales	NC_014570.1	Paiva *et al.* (2011)
37	*Eugenia uniflora*	Myrtaceae	Myrtales	NC_027744.1	Eguiluz *et al.* (2017)
38	*Allomaeita villosa*	Melastomataceae	Myrtales	KX826819	Reginato *et al.* (2016)
39	*Bertolonia acuminate*	Melastomataceae	Myrtales	KX826820	Reginato *et al.* (2016)
40	*Graffenrieda moritziana*	Melastomataceae	Myrtales	KX826823	Reginato *et al.* (2016)
41	*Henriettea barkeri*	Melastomataceae	Myrtales	KX826824	Reginato *et al.* (2016)
42	*Miconia dodecandra*	Melastomataceae	Myrtales	KX826826	Reginato *et al.* (2016)
43	*Nepsera aquatica*	Melastomataceae	Myrtales	KX826827	Reginato *et al.* (2016)

	Taxon	Family	Order	Accesion number	Study
44	*Rhynchanthera bracteata*	Melastomataceae	Myrtales	KX826831	Reginato *et al.* (2016)
45	*Oenothera argillicola*	Onagraceae	Myrtales	NC_010358.1	Greiner *et al.* (2008)
46	*Oenothera biennis*	Onagraceae	Myrtales	NC_010361.1	Greiner *et al.* (2008)
47	*Oenothera elata subsp.hookeri*	Onagraceae	Myrtales	NC_002693.2	Hupfer *et al.* (2000)
48	*Oenothera glazioviana*	Onagraceae	Myrtales	NC_010360.1	Greiner *et al.* (2008)
49	*Oenothera parviflora*	Onagraceae	Myrtales	NC_010362.1	Greiner *et al.* (2008)
50	*Stockwellia quadrifida*	Myrtaceae	Myrtales	NC_022414.1	Bayly *et al.* (2013)
51	*Syzygium cumini*	Myrtaceae	Myrtales	GQ870669.3	Asif *et al.* (2013)
52	*Acer buergerianum sub ningpoense*	Aceraceae	Sapindales	KF753631.1	Yang *et al.* (2014)
53	*Azadirachta indica*	Meliaceae	Sapindales	NC_023792.1	unpublished
54	*Citrus sinensis*	Rutaceae	Sapindales	NC_008334.1	Bausher *et al.* (2006)
55	*Sapindus mukorossi*	Sapindaceae	Sapindales	NC_025554.1	Yang *et al.* (2014)
56	*Vitis vinifera*	Vitaceae	Vitales	NC_007957.1	Jansen *et al.* (2006)

Sato S, Nakamura Y, Kaneko T, Asamizu E and Tabata S (1999) Complete structure of the chloroplast genome of *Arabidopsis thaliana*. DNA Res 6:283-90.

Hu, ZY, Hua W, Huang SM and Huang HZ (2011) Complete chloroplast genome sequence of rapeseed (*Brassica napus* L.) and its evolutionary implications. Genet Resour Crop Evol 58:875-887.

Jeong YM, Chung WH, Mun JH, Kim N and Yu HJ (2014) De novo assembly and characterization of the complete chloroplast genome of radish (*Raphanus sativus* L.). Gene 551:39-48.

Weng ML, Blazier JC, Govindu M and Jansen RK (2014) Reconstruction of the Ancestral Plastid Genome in Geraniaceae Reveals a Correlation between Genome Rearrangements, Repeats, and Nucleotide Substitution Rates. Mol Biology and Evol 31:645-659.

Blazier JC, Guisinger MM and Jansen RK (2011). Recent loss of plastid-encoded ndh genes within *Erodium* (Geraniaceae). Plant Mol Biol 76:263-272.

Guisinger MM, Kuehl JV, Boore JL and Jansen RK (2011). Extreme reconfiguration of plastid genomes in the angiosperm family Geraniaceae: Rearrangements, repeats, and codon usage, Mol Biol Evol 28:583-600.

Tangphatsornruang S, Uthaipaisanwong P, Sangsrakru D, Chanprasert J, Yoocha T, Jomchai N and Tragoonrung S (2011) Characterization of the complete chloroplast genome of *Hevea brasiliensis* reveals genome rearrangement, RNA editing sites and phylogenetic relationships. Gene 475:104-12.

Asif MH, Mantri SS, Sharma A, Srivastava A, Trivedi I, Gupta P, Mohanty CS, Sawant SV and Tuli R (2010) Complete sequence and organisation of the *Jatropha curcas* (Euphorbiaceae) chloroplast genome. Tree Genetics and Genomes 6:941-952.

Jansen RK, Saski C, Lee SB, Hansen AK and Daniell H (2011) Complete plastid genome sequences of three rosids (*Castanea*, *Prunus*, *Theobroma*): Evidence for at least two independent transfers of rpl22 to the nucleus. Mol Biology and Evol 28:835-847.

Machado LO, Vieira LD, Stefenon VM, Pedrosa OF, De Souza EM, Guerra MP and Nodari RO (2017) Phylogenomic relationship of feijoa (*Acca sellowiana* (O. Berg) Burret) with other Myrtaceae based on complete chloroplast genome sequences. Genetica 145:1-12.

Bayly MJ, Rigault P, Spokevicius A, Ladiges PY, Ades PK, Anderson C, Bossinger G, Merchant A, Udovicic F, Woodrow IE, *et al.* (2013) Chloroplast genome analysis of Australian

eucalypts - Eucalyptus, Corymbia, Angophora, Allosyncarpia and Stockwellia (Myrtaceae). Mol Phylogenet Evol 69:704-716.

Paiva JA, Prat E, Vautrin S, Santos MD, San-Clemente H, Brommonschenkel S, Fonseca PG, Grattapaglia D, Song X, Ammiraju JS, *et al.* (2011) Advancing Eucalyptus genomics: Identification and sequencing of lignin biosynthesis genes from deep-coverage BAC libraries. BMC Genomics 12:137.

Eguiluz M, Rodrigues FN, Guzman F, Yuyama P and Margis R (2017) The chloroplast genome sequence from *Eugenia uniflora*, a Myrtaceae from Neotropics. Plant Syst Evol 1-14.

Reginato M, Neubig KM, Majure LC and Michelangeli FA (2016) The first complete plastid genomes of Melastomataceae are highly structurally conserved. Peer J 4:e2715.

Greiner S, Wang X, Rauwolf U, Silber MV, Mayer K, Meurer J, Haberer G and Herrmann RG (2008) The complete nucleotide sequences of the five genetically distinct plastid genomes of Oenothera, subsection Oenothera: I. sequence evaluation and plastome evolution. Nucleic Acids Res 36:2366-2378.

Hupfer H, Swaitek M, Hornung S, Herrmann RG, Maier RM, Chiu WL, *et al.* (2000) Complete nucleotide sequence of the *Oenothera elata* plastid chromosome, representing plastome 1 of the five distinguishable Euoenthera plastomes. Mol Gen Genet 263:581-585.

Asif H, Khan A, Iqbal A, Khan IA, Heinze B and Azim MK (2013) The chloroplast genome sequence of *Syzygium cumini* (L.) and its relationship with other angiosperms. Tree Genet Genomes 9:867-877.

Yang JB, Li DZ and Li HT (2014) Highly effective sequencing whole chloroplast genomes of angiosperms by nine novel universal primer pairs. Mol Ecol Resour 14:1024-1031.

Bausher MG, Singh ND, Lee SB, Jansen RK and Daniell H (2006) The complete chloroplast genome sequence of *Citrus sinensis* (L.) Osbeck var 'Ridge Pineapple': Organization and phylogenetic relationships to other angiosperms. BMC Plant Biol 6:21.

Jansen RK, Kaittanis C, Saski C, Lee SB, Tomkins J, Alverson AJ *et al.* (2006). Phylogenetic analyses of *Vitis* (Vitaceae) based on complete chloroplast genome sequences: Effects of taxon sampling and phylogenetic methods on resolving relationships among rosids. BMC Evol Biol 6:32.
